# Effect of Microstructure on Micro-Mechanical Properties of Composite Solid Propellant

**DOI:** 10.3390/mi12111378

**Published:** 2021-11-10

**Authors:** Tianpeng Li, Jinsheng Xu, Junli Han, Yong He

**Affiliations:** 1 School of Mechanical Engineering, Nanjing University of Science and Technology, Nanjing 210094, China; ltp820325@163.com (T.L.); xujinsheng@njust.edu.cn (J.X.); yhe1964@njust.edu.cn (Y.H.); 2 Shijiazhuang Campus, Army Engineering University, Shijiazhuang 050003, China; 3 Beijing Institute of Special Electromechanical Technology, Beijing 100012, China

**Keywords:** composite solid propellant, microstructure model, numerical simulation, mechanical property

## Abstract

This study was aimed at determining the effect of microstructure on the macro-mechanical behavior of a composite solid propellant. The microstructure model of a composite solid propellant was generated using molecular dynamics algorithm. The correlation of how microstructural mechanical properties and the effect of initial interface defects in propellant act on the macro-mechanics were studied. Results of this study showed that the mechanical properties of propellant rely heavily on its mesoscopic structure. The grain filling volume fraction mainly influences the propellant initial modulus, the higher the volume fraction, the higher initial modulus. Additionally, it was found that the ratio of particles influences the tensile strength and breaking elongation rate of the propellant. The big particles could also improve the initial modulus of a propellant, but decrease its tensile strength and breaking elongation rate. Furthermore, the initial defects lowered the uniaxial tensile modulus, tensile strength, and the relaxation modulus of propellant, but did not affect the relaxation behavior of the propellant.

## 1. Introduction

Composite solid propellant is a high-energy composite material that is widely used as a power source for launch vehicles and various strategic and tactical missiles. The mechanical properties of the composite solid propellant greatly affect the survivability and combat capability of the missile. Moreover, composite solid propellant is composed of the hydroxyl terminated polybutadiene (HTPB) as a binder matrix and solid particles such as aluminum powder (AL), ammonium perchlorate (AP), and hexogen (RDX) as filler. The macro mechanical properties of composite solid propellant strongly depend on the mesostructure and its multi-scale physical process under an external load. Early research on composite solid propellants is mostly based on continuum mechanics to obtain the constitutive relationship of the composite solid propellant [1]. However, this method cannot effectively expose the internal structure change mechanism of the composite solid propellant. Therefore, it is necessary to study the structural characteristics of the composite solid propellant from a mesoscopic perspective.

Numerous experimental investigations have been conducted in recent years to explore the micromechanical properties and failure mechanisms of composite solid propellants. D. Bencher [2] and Liu C T [3] analyzed the crack formation through experimental method. Marthinus C J [4] studied the microstructure deformation and fracture behavior of a hydroxyl-terminated polybutadiene (HTPB) propellant by using in-situ uniaxial tensile experiments conducted on a scanning electron microscope (SEM). Above all, the experimental methods explore the particle–matrix interfacial debonding as the main source of failure of the composite solid propellant [5,6].

With the development of computational technologies, Matouš and Inglis [7] develop the micromechanics model with the finite element mesh division. This is in terms of studying the meso-damage of the propellant, the failure and failure mechanism of the bonding interface, as well as the internal causes of macro mechanical properties of propellant. The study by Matouš and Inglis also set the bonding elements in the interface layer between the particles and matrix to simulate the generation and development of interface dehumidification damage. This showed that the interfacial debonding is the main reason for the macroscopic stress-strain nonlinearity of the propellant. On this basis, Chang [8] found that the interface damage is closely related to the size and relative position of particles. H. Arora [9] simulated the deformation and damage evolution process of polymer-bonded explosives. It was found that the particle geometries had a great influence on the onset of failure.

The mechanical properties of the bonding interface between the particles and matrix are important factors that could affect the macro stress-strain relationship of propellant. Based on the characteristics of meso-damage of propellant, Li [10] introduced the bonding interface element between particle and matrix and described the propagation characteristics of interface damage by a bilinear cohesion model. They studied the interface debonding process of propellant and its influence on macro mechanical response through finite element calculation. An approximate characterization of the mechanical response of propellant particle/matrix interface by bilinear cohesion model was carried out by Zhi [11]. It was found that the initial modulus and tensile strength of propellant increased with the increase of the filling volume fraction. Further, the random distribution of particle position hardly affected its mechanical properties.

Elsewhere, Han [12] found that the bilinear cohesion model is not accurate enough to reflect the interface mechanical behavior of real propellant. However, it was evident that the rate dependent exponential cohesion model can accurately simulate the crack propagation process of HTPB propellant under mixed loading mode. Cui [13] proposed a novel time-dependent cohesive zone material (CZM) based on the Maxwell box to simulate relaxation responses. Based on their studies, Ahmad [14] and Zhi [15] compared the stress-strain curve obtained by numerical simulation with the test curve to establish the optimization objective function of damage parameters. The dehumidification damage parameters obtained through step-by-step iterative calculation were used to simulate the meso-damage process of propellant and the results were in consonance with the macro test.

These described studies considered that the mesoscopic composition of propellant is intact but various forms of initial defects existing in the production process of propellant were not considered. The existence of these defects may not only affect the macro mechanical properties of propellant but also affects the combustion characteristics during engine ignition. There are few studies on the effect of initial defects on the mechanical properties of propellant. It has been reported that He [16] studied the effects of cracks, bubbles, and bonding defects in propellant on its combustion performance. Elsewhere, Du [17], Erkkilä, [18] and Xiao [19] regard concrete as a multiphase heterogeneous composite composed of mortar, aggregate, interface, and defects. Through numerical research, it was found that the distribution position of initial defects has fewer effects on concrete strength, but has an obvious effect on tensile strength. Therefore, the influence of internal defects should not be ignored in the study of the mechanical properties of composites. In this study, the effects of mesoscopic structures on the macro mechanical properties of propellant such as particle volume fraction, particle size, and initial defects were evaluated using mesoscopic finite element numerical calculation method.

## 2. Construction of Singular Crack Element

### 2.1. Mesoscale Model of Composite Solid Propellants

The formula and component information of a composite solid propellant were shown in Table 1.

**Figure 1:**
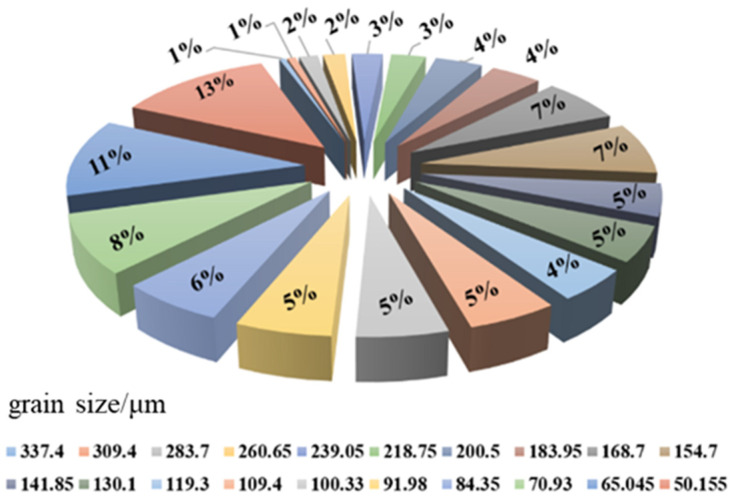
Particle size distribution of hydroxyl terminated polybutadiene (HTPB) propellant.

It was found that the number ratio of different particles is related to their corresponding particle size. According to the size distribution of AP particles in propellant obtained through a real test given in the literature [20], the number fraction of particles with corresponding size can be calculated, as shown in Figure 1. Furthermore, the mesoscopic particle filling model of HTPB propellant can also be established (Figure 1).

**Figure 2:**
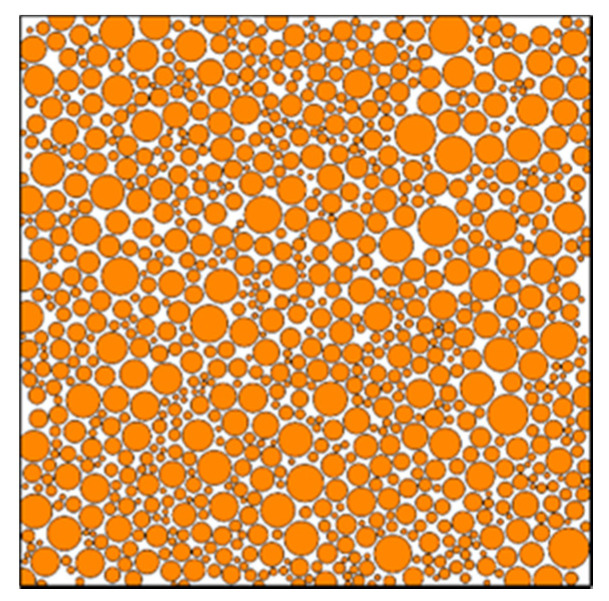
Microparticle filling model of HTPB composite solid propellant.

The mesoscopic particle filling model of composite solid propellant was generated based on the molecular dynamics algorithm program as shown in Figure 2. This was combined with the typical propellant formula components in Table 1 and the filling particle size distribution law in Figure 1. It was found that the number of particles filled was 1000 and the filling volume fraction was 76%, whereas the actual representative volume element (RVE) size was 4410 μm^2^ × 4410 μm^2^. Similar to the experimental observation results, it was found that the particles were randomly and evenly distributed, closely staggered, while the small particles were distributed in the gap of large particles (Figure 2). The small size of Al particles led to a large number of particles, which also led to the phenomenon of particle overlap during the generation of the model. Furthermore, it is not easy to calculate the later finite element method. Therefore, this study adopted a filling model in which Al particles are regarded as a part of the matrix and only larger AP particles are considered.

### 2.2. Simulation Parameters of the Meso-Mechanical Model

The elastic modulus of AP particles is much larger than that of the substrate. Therefore, it can be regarded as an elastomer in finite element calculation. The main parameters in this study were *E*_AP_ = 32,447 MPa and *ν*_AP_ = 0.1433. However, other parameters were as listed in Table 2. The equivalent matrix can be regarded as a polymer with both hyperelasticity and viscoelasticity and its mechanical properties are characterized by the viscoelastic constitutive model;

$$\begin{array}{l} \sigma =\sigma^{e}+\sigma^{v}\\ \sigma^{e}=\sum_{n=1}^{2}\frac{2\mu_{n}}{\alpha_{n}}\left[\lambda^{\alpha_{n}-1}-\lambda^{-\alpha_{n}/2-1}\right]\\ \sigma^{v}=\int_{0}^{t}\sum_{m=1}^{2}E_{m}\exp\left(-\frac{t-\tau }{\tau_{m}}\right)\frac{\partial \varepsilon }{\partial \tau }d\tau \end{array}$$

The bonding interface between propellant particles and matrix was described by the bilinear cohesion model. The interface was idealized as the thickness free surface with certain mechanical properties such as bonding strength. The mechanical response of the bonding element is defined by the traction displacement law, so as to characterize the damage initiation and evolution at the whole interface. The parameters of the cohesion model were obtained by inverse optimization method, and the specific parameters are shown in Table 3.

## 3. Effect of Mesostructure on Mechanical Properties of Propellant

### 3.1. Influence of Particle Volume Fraction

**Figure 3:**
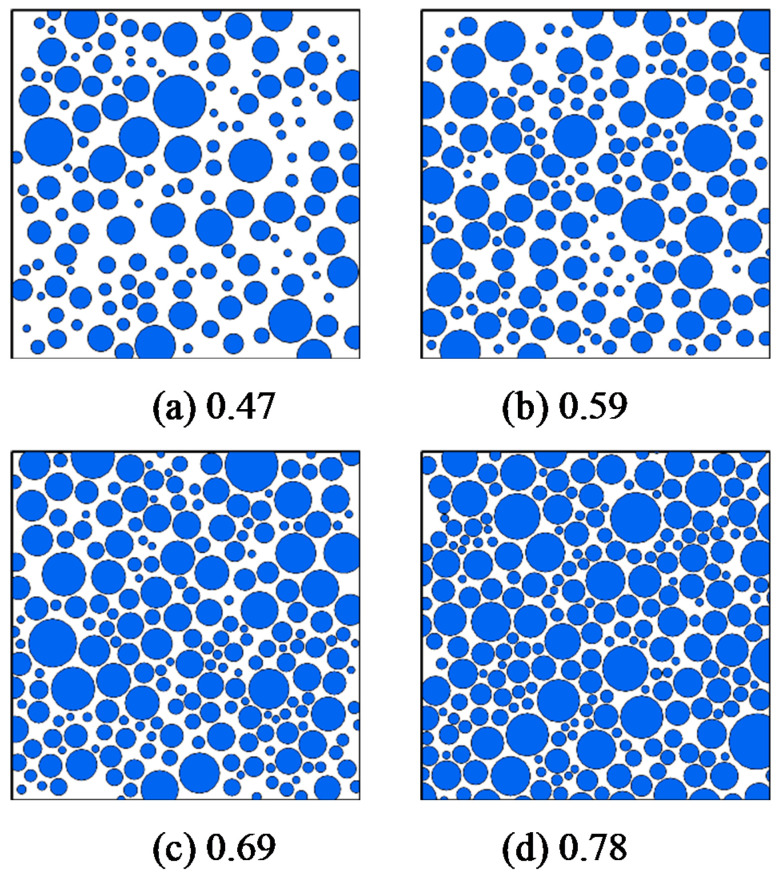
Mesoscale propellant filling models with different particle volume fractions.

Four groups of propellant mesoscopic filling models with different filling volume fractions were established to explore the influence of different propellant particle filling volume fractions on their macro mechanical properties in this study (Figure 3). The corresponding particle volume fractions of the model were 0.47, 0.59, 0.69, and 0.78 (Figure 3).

**Figure 4:**
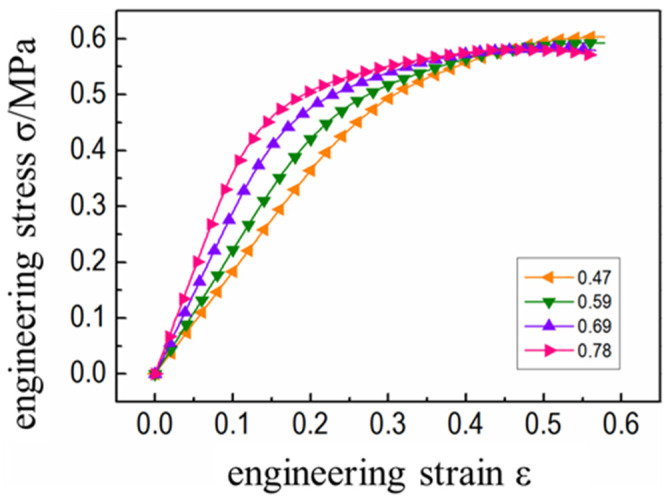
Stress-strain curves of propellants with different volume fractions.

In this study, the meso-mechanical model of HTPB propellant with different filling volume fractions was numerically calculated. The stress-strain curve of the corresponding calculation results is shown in Figure 4. It was found that the initial slope of the tensile stress-strain curve of propellant with different particle volume fractions is quite different (Figure 4). The larger the particle volume fraction, the greater the curve slope and the earlier that the curve entered the nonlinear section. This was because the particle volume fraction significantly determined the initial modulus of the propellant, which determined the time when the mesoscopic stress in the propellant reached the critical value of interface damage. Further, it was found that the larger the volume fraction of filled particles, the greater the initial modulus of the propellant and the faster the internal mesoscopic stress transfer. The stress at the particle/matrix interface reached the damage critical value at the earliest and the “dehumidification” damage began to appear. Therefore, the corresponding stress-strain curve entered the nonlinear section at the earliest until the final fracture failure of the propellant. It was also evident that after propellants with different particle volume fractions entered the stress “platform area”, the volume fraction increased whereas the degree of stress declined (Figure 4). This indicates that the mechanical properties of HTPB propellant with higher volume fractions decline more violently due to “dehumidification”, which is consistent within the previous literature [21].

### 3.2. Influence of Multi-Particle Gradation

The mesoscopic filling model of propellant with different particle grading was established to study the effect of different particle sizes on the mechanical properties of HTPB propellant. The selection of AP particle size was based on the commonly used values in the current project and was combined accordingly. The specific particle grading information is as shown in Table 4. To eliminate the influence of particle volume fraction, the corresponding particle volume fraction of the four particle filling models with different gradations was set at 0.69.

**Figure 5:**
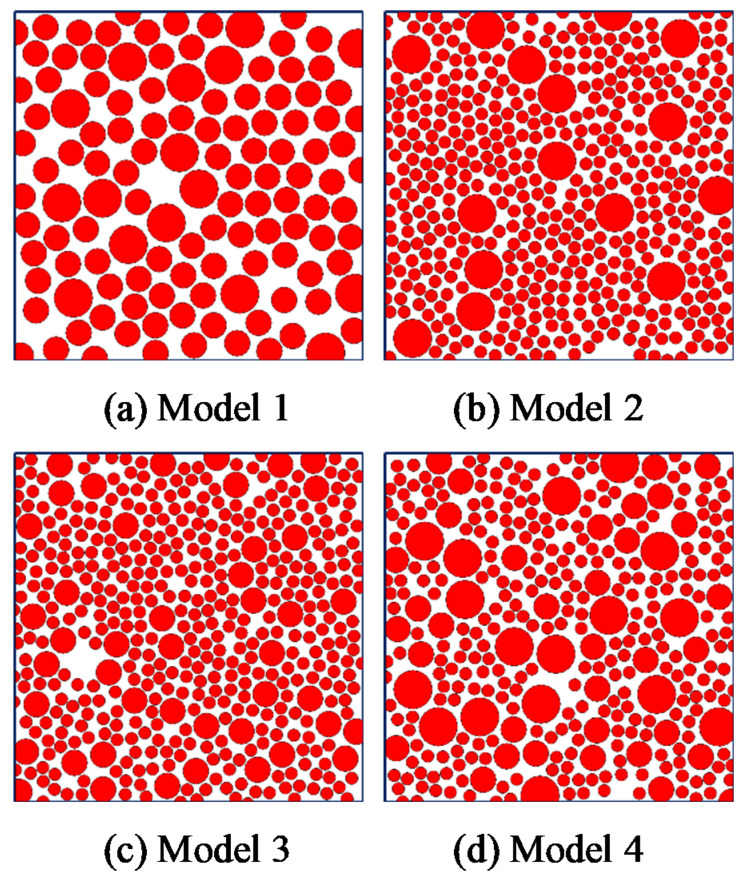
Mesoscale filling model of propellant with different particle sizes.

According to the ratio results (Table 4), the final four mesoscopic filling models of propellants with different particle gradations are shown in Figure 5. The different particle gradations finally show meso-mechanical models with different filling fullness (Figure 5). Model 1, containing only AP particles with larger particle size, was sparsely filled and was not dense enough. In the model with a small particle size, the particles were embedded in the gap between large particles, the filling structure was closer, and the filling performance was better than the model with larger particles.

**Figure 6:**
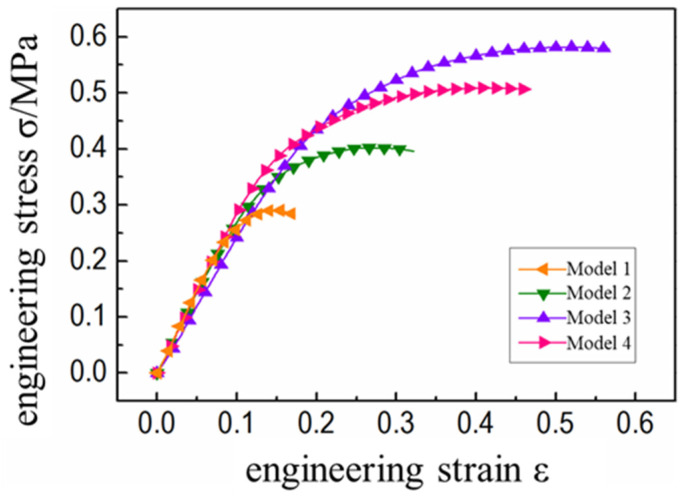
Stress-strain curves of propellants with different particle sizes.

The stress-strain curves of HTPB propellant with different particle gradations were as shown in Figure 6. It was observed that on the premise of a certain particle volume fraction, the initial modulus of propellant was not affected by particle gradation. Only Model 3 composed of small particle size particles had a slightly lower initial modulus than the other three graded propellants, as shown Table 5. This shows that the enhancement effect of larger particle size on the mechanical properties of propellant is more obvious. The difference in mechanical properties of graded propellants is mainly reflected in the nonlinear section.

The propellant with the large particle size formula reached the stress peak first because under the same load, the “dehumidification” damage is more likely to occur at the interface between large particles and matrix, resulting in the decrease of propellant stress value. For the meso-mechanical model of propellant with large average particle size and grading, the degree of particle “dehumidification” in the tensile process was more important and resulted in a decline of the mechanical properties of the propellant. Models 1, 2, and 4 contained large particle size particles. When bearing the load, the corresponding propellant first produced macro cracks due to the continuous convergence of micropores left by larger particle interface debonding, resulting in propellant failure and reduced propellant elongation (Table 5).

## 4. Effect of Initial Defects on Mechanical Properties of Propellant

**Figure 7:**
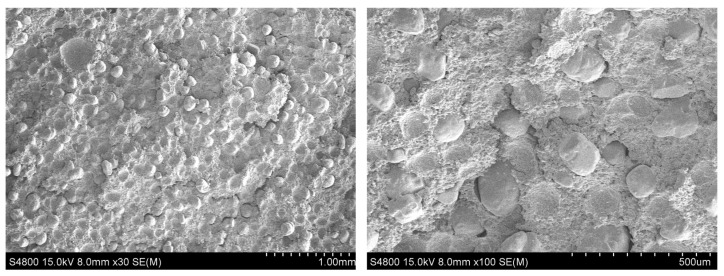
SEM image of initial section of HTPB propellant.

The scanning electron microscope photograph of the initial section of HTPB propellant (Figure 7) revealed that there were many initial defects in the propellant. There is no perfect package between the particles and the matrix. The initial defects are formed when the propellant is stirring, curing, and cooling as well as the vacuum degree.

### 4.1. Definition and Modeling of Initial Interface Defects

The macroscopic mechanical test of propellant confirmed that [22] the particle/matrix bonding interface in the propellant is the weakest link in its structure and the debonding of the interface under load is the root cause of propellant failure. At the same time, it was found that the main form of propellant initial defects is particle/matrix interface bonding defects generated during curing and cooling (Figure 7). Therefore, only the influence of the initial defects at the propellant particle/matrix interface on its macro mechanical properties was considered in this study.

The following assumptions are made for the interface defects:

1. The initial defects are uniformly and randomly distributed in the interface element.
2. For the defect interface, the failure bonding element is used to simulate.
3. Define the interface defect content as *p*, *p* = *N_d_*/*N*. *N* is the total number of interface units and *N_d_* is the total number of defective units.

**Figure 8:**
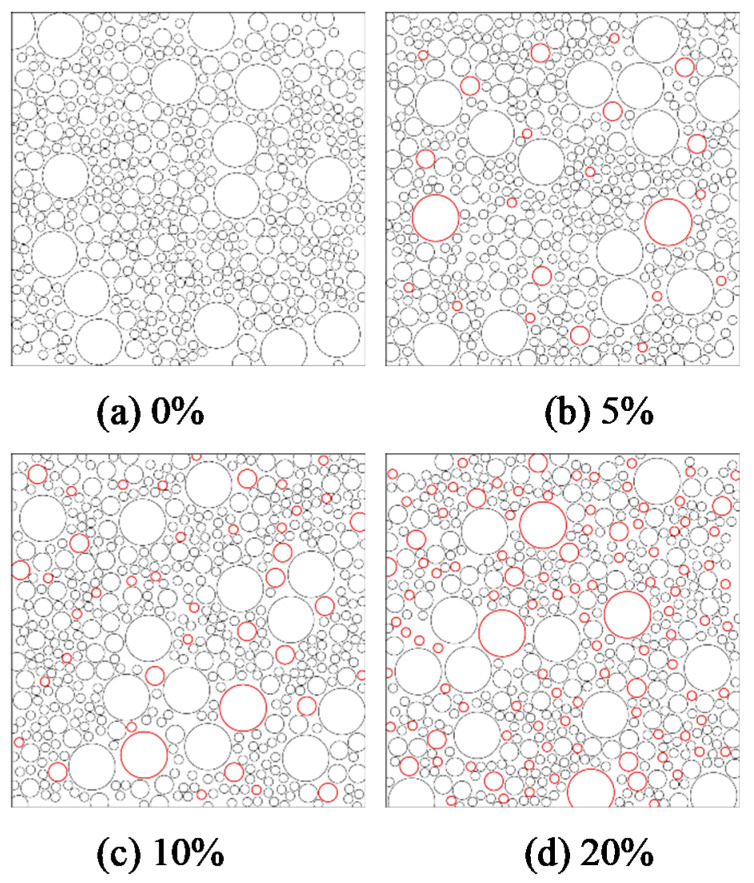
Mesoscale model of propellant with different interface defect contents.

To study the influence of mesoscopic interface bonding defects on its macro mechanical properties, it is necessary to establish a propellant mesoscopic particle filling model with interface defects [23]. During modeling, the mesoscopic component parameters selected the propellant meso-mechanical model 4 as the object and established four groups of models with initial interface defect contents of 0%, 5%, 10%, and 20% as shown in Figure 8.

### 4.2. Effects of Initial Interface Defects on Tensile Mechanical Properties of the Propellant

**Figure 9:**
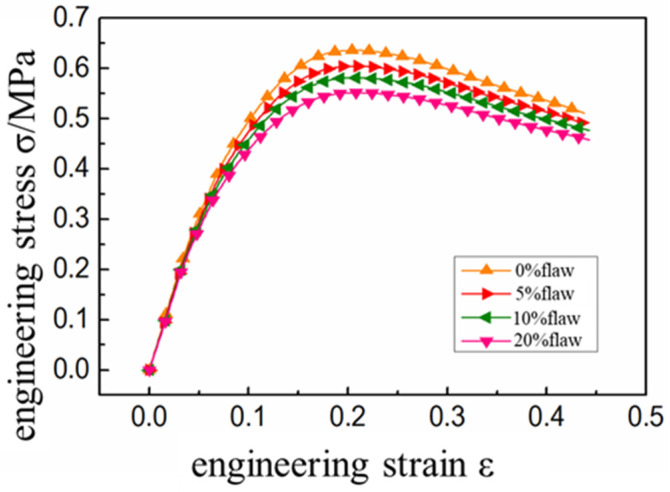
Uniaxial tensile results of HTPB propellant with initial interface defects.

An isokinetic displacement load with a rate of 20 mm/min was applied to the model boundary. The interface mechanical property parameters at this rate are brought in to simulate the mechanical response of the propellant. The main mechanical property parameters of the propellant were obtained through calculation and the specific parameters were as shown in Table 5. Further, the stress-strain curve was as shown in Figure 9.

It was evident that the existence of interface defects reduces the initial modulus of the propellant, and the higher the defect content, the more intense the modulus decreases (Figure 9). This is because the defect interface is defined as a failure element, which does not transfer load during tension [24,25]. Therefore, at the initial stage of load, the model with more initial defects has worse load bearing capacity. The corresponding stress value is lower under the same strain level. The existence of the initial defects reduces the tensile strength of the composite solid propellant. This was mainly because the interface defects were always in the failure state and the particles at the interface cannot play the enhancement effect [26]. This obviously reduces the overall strength of the propellant and the higher the defect content, the lower the tensile strength of the propellant.

**Figure 10:**
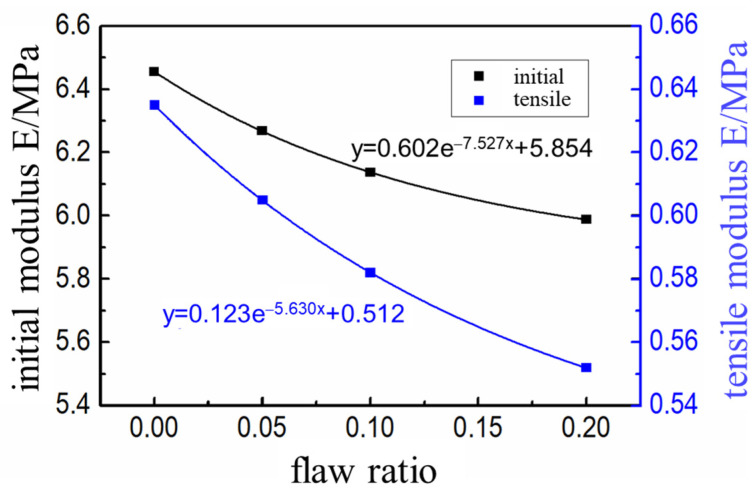
The variation of initial modulus and tensile strength with the interface defect content.

According to the initial modulus *E*_in_ and tensile strength *σ*_m_ of the propellant with interface defect content in Table 6, an exponential relationship was established. The exponential function was selected to fit the relationship and the corresponding fitting results were as shown in Figure 10.

### 4.3. Effects of Initial Interface Defects on Mechanical Properties of Propellant Relaxation

**Figure 11:**
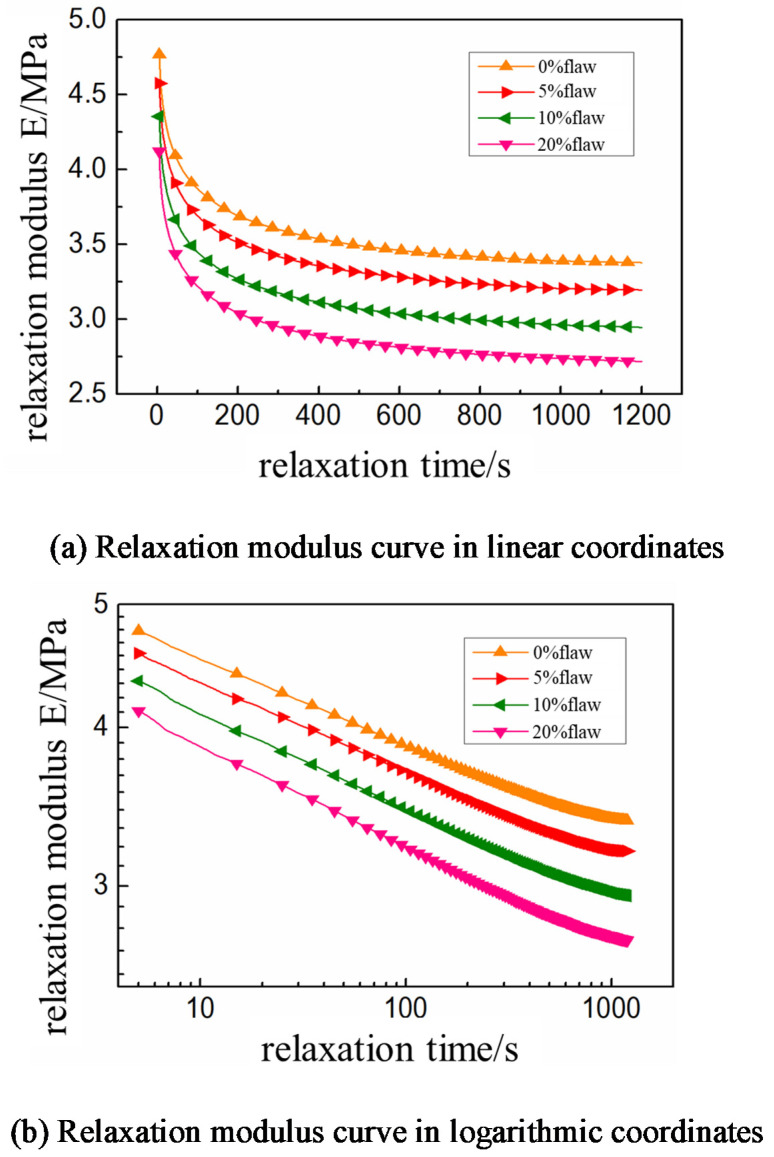
Stress relaxation results of HTPB propellant with initial interface defects.

The boundary conditions are applied in two steps. The first analysis step is to apply a constant displacement load of 100 mm/min on the boundary of the model. This is to stretch the model to 10% strain. The second analysis step is to keep the displacement of the upper boundary of the model unchanged. It also involves to output the time-varying results of the force on the boundary of the model in the next 1200 s [27] and calculate the time-varying relationship of the modulus of the propellant, as shown in Figure 11.

It can be seen from Figure 11 that the values of stress relaxation curves of HTPB propellant with different interface defect contents are different. However, the overall trend is the same, and the higher the content of interface defects, the lower the relaxation modulus of the propellant. It shows that the interface defects in the propellant only affect the relaxation modulus, not the relaxation rate, because the relaxation characteristics of the composite solid propellant are determined by the properties of the matrix material, which is irrelevant with the initial defects during the preparation process [28].

## 5. Conclusions

In this study, the finite element numerical calculation of HTPB propellant models with different mesoscopic structures was evaluated. The correlation between the mechanical properties of the propellant and its mesoscopic structure was analyzed and the influence of the existence of initial interface defects in the propellant on its macro tensile and stress relaxation characteristics were studied. The results of this study showed that: (1) The mechanical properties of HTPB propellant depend heavily on its mesoscopic structure and the random distribution of particles hardly affects its macro mechanical properties. The simulation results present the initial modulus of the composite solid propellant increases with the increase of the particle filling volume fraction. The different particle ratio significantly affects the tensile strength and fracture elongation of the propellant. Although the existence of large particles improves the initial modulus of the propellant, it reduces its tensile strength and fracture elongation. (2) The main forms of initial defects were analyzed using a scanning electron microscope of the initial section of HTPB propellant. The filling models with different initial interface defect contents are constructed to predict the uniaxial tension and stress relaxation process. It was found that the initial defect is the main reason for the decrease of the initial modulus, relaxation modulus, and tensile strength of the composite solid propellant. However, the relaxation characteristics of the composite solid propellant will not disappear with the increase of the initial defects.

## Figures and Tables

**Table 1:** Typical composition of HTPB propellant.

Component	Adhesive	AP	Al	Other Components
mass fraction (%)	8.0	69.5	18.5	4
density (g/cm^3^)	0.90	1.95	2.70	-
volume fraction (%)	23.9	63.8	12.3	-

**Table 2:** Viscoelastic model parameters of HTPB propellant matrix film.

*μ*_1_/MPa	*α*_1_	*μ*_2_/MPa	*α*_2_	*E*_1_/MPa	*τ*_1_/s	*E*_2_/MPa	*τ*_2_/s
0.235	0.525	7.529 × 10^−5^	6.705	0.0552	29.319	0.0216	392.201

**Table 3:** AP interface mechanical model parameters obtained based on stepwise inversion analysis.

Rate of Extension (mm·min^−1^)		1	5	20	100	500
AP-1 (250–300 μm)	$K/\mathrm{MPa}\cdot {\mathrm{mm}}^{-1}$	550	610	680	760	860
	$T^{\max}/\mathrm{MPa}$	0.346	0.454	0.586	0.723	0.831
	$\delta^{f}/\mathrm{mm}$	0.012	0.012	0.012	0.012	0.012
AP-2 (100–150 μm)	$K/\mathrm{MPa}\cdot {\mathrm{mm}}^{-1}$	520	580	650	720	800
	$T^{\max}/\mathrm{MPa}$	0.382	0.472	0.604	0.756	0.862
	$\delta^{f}/\mathrm{mm}$	0.015	0.015	0.015	0.015	0.015
AP-3 (10–20 μm)	$K/\mathrm{MPa}\cdot {\mathrm{mm}}^{-1}$	500	560	620	700	780
	$T^{\max}/\mathrm{MPa}$	0.442	0.558	0.683	0.779	0.921
	$\delta^{f}/\mathrm{mm}$	0.018	0.018	0.018	0.018	0.018

**Table 4:** Particle size distribution of propellants at different gradations.

Model	1	2	3	4
grain size/μm	246-165	246-80	165-80	246-165-80
volume ratio	3:7	3:7	3:7	1:1:1

**Table 5:** Mechanical property of propellants at different gradations.

Model	1	2	3	4
Initial modulus/MPa	2.84	2.80	2.41	2.88
Elongation	0.17	0.32	0.46	0.57

**Table 6:** Mechanical property parameters of HTPB propellant containing interface defects.

Performance Index	Parameter			
Defect ratio (%)	0	5	10	20
Initial modulus (MPa)	6.456	6.268	6.137	5.988
Tensile strength (MPa)	0.635	0.605	0.582	0.552
